# United States Veterinary Medical Association

**Published:** 1890-10

**Authors:** 


*United States Veterinary Medical Association, Twenty-seventh Annual Meeting, September 16th and 17th, 1890.*

OFFICERS ELECTED FOR ENSUING YEAR.

*President*, Dr. R. S. Huidekoper, Philadelphia; *Vice-President*, Dr.

W. L. Williams, Bloomington, Ill.; *Secretary*, Dr. W. H. Hoskins, 12 S. Thirty-seventh Street, Philadelphia; *Treasurer*, Dr. James L. Robertson, New York city.

OFFICERS AND COMMITTEES APPOINTED FOR ENSUING YEAR.

*Board of Censors.*—Dr. W. J. Coates. New York, Chairman; Dr. J. F. Winchester, Massachusetts; Dr. Tait Butler, Iowa; Dr. C. C. Lyford, Minnesota; Dr. R. A. McLean, New York; Dr. Thomas B. Rayner, Pennsylvania; Dr. William Dougherty, Maryland.

*Committee on Intelligence and Education.*—Dr. Austin Peters, Chairman; Dr. Withers, Dr. W L. Zuill, Dr. P. Paquin, Dr. Gerald B. Griffin.

*Committee on Finance.*—Dr. Charles Burden, Chairman; Dr. D. J. Dixon, Dr. E. C. Ross.

*Committee on Diseases.*—Dr. Tait Butler, Iowa, Chairman; Dr. Charles B. Michener, New York; Dr. James L. Kidd, Kentucky; Dr. James A. Waugh, 6th U. S. Cavalry; Dr. George Bridges, Connecticut.

*Committee on Prizes.*—Dr. D. E. Salmon, Washington, D. C., Chairman; Dr. Dixon, New Jersey; Dr. E. C. Ross.

*Special College Committee.*—Dr. C. C. Lyford, Minnesota, Chairman; Dr. C. S. Breed, Massachusetts; Dr. A. W. Clement, Maryland.

*Committee on Army Legislation.*—Dr. W. B. E. Miller, New Jersey, Chairman; Dr. D. Lemay, Kansas; Dr. Cooper Curtice, D. C.

*Committee on Publication.*—Dr. W. Horace Hoskins, Pennsylvania, Chairman; Dr. C. T. Goentner, Pennsylvania; Dr. S. E. Weber, Pennsylvania.

*Special Committee to Consider Plan for Central Veterinary Body.*—Dr. C. P. Lyman, Massachusetts, Chairman; Dr. James L. Robertson, New York; Dr. G. H. Berns, New York.

*Special Committee on Food Inspection.*—Dr. W. L. Williams, Dr. Olof Schwartzkopff, Dr. A. W. Clement.

LIST OF NEW MEMBERS ELECTED WITH VOUCHERS.

Roscoe R. Bell, D.V.S., Am. Vet. Coll. (Dr. R. A. McLean), 7th Ave., and Union Street, Brooklyn, N. Y.; Thomas M. Buckley, D.V.S., Am. Vet. Col. (Dr. R. A. McLean) 480 Clermont Ave., Brooklyn, N.Y.; Gerald E. Griffin, D.V.S., Am. Vet. Coll. (Dr. W. J. Coates), 5th Cavalry, Fort Reno, Ind. Ty.; Richard R. Morrison, D.V.S., Am. Vet. Coll. (Dr. W. J. Coates), 141 West Fifty-fourth Street, New York City; Joseph Ogle, Jr., D.V.S., Am. Vet. Coll. (Dr. W. J. Coates), 302 W. Forty-sixth Street, New York City; R. W. Hickman, V.M.D., Vet. Dept. U. of Pa. (Dr. C. B. Michener). Green Ave. and Broadway, Brooklyn, N. Y.; H. B. Ambler, D.V.S., Am. Vet. Coll. (Dr. C. B. Michener), Green Ave. and Broadway, Brooklyn, N. Y.; J. L. Kilborne, B.V.S., Cornell Vet. Dept. (Dr. J. F. Winchester), Dpt. of Agriculture, Washington, D. C.; Richard Letts, D.V.S., Am. Vet. Coll. (Dr. W. B. E. Miller), Bloomfield Street, Hoboken, N. J.; D. S. Breslin, D.V.S., Am. Vet. Coll. (Dr. W. H. Pendry), 94 Adams Street, Brooklyn, N. Y.; A. J. Thompson, D.V.S., Am. Vet. Coll. (Dr. W. Horace Hoskins), Evansville, Ind.; J. Huhne, D.V.S., Am. Vet. Coll. (Prof. A. Liautard), 141 West Fifty-fourth Street, New York City; R. G. Webster, V.M.D., Vet. Dpt U. of Pa.,

(Dr. W. Horace Hoskins), Media, Pa.; Harry E. Bates, D.V.S., Am. Vet. Coll. (Dr. E. C. Ross), New Haven, Conn.; T. L. Armstrong, D.V.S., Chi. Vet. Coll. (Dr. W. H. Wray), Hotel Richmond, Chicago, Ill.; N. P. Valerius, D.V.S., Am. Vet. Coll., Watertown, Wis.; James A. Waugh, V.S., (Dr. H. D. Gill), 6th Cavalry, U. S. A., Fort Wingate, N.M.; E. A. A. Grange, V.S., Toronto Vet. Coll. (Dr. Tait Butler), Agricultural College, Mich.; John Tillie, D.V.M., Iowa Agril. Coll. (Dr. Tait Butler), Muscatine, Iowa; S. Stewart, M.D., D.V.M., (Dr. Tait Butler), Council Bluffs, Iowa.; C. A. Carey, B.S., D.V.M., Iowa Agr. Coll. (Dr. Tait Butler), Brookings, South Dakota; G. C. Williams, V.S., Ont. Vet. Coll. (Dr. Tait Butler), Dewitt, Iowa; John D. Rutherford, V.S., Ont. Vet. Coll (Dr. Tait Butler), Rock Island, Ill.; George A. Johnson, D.V.M., Vet. Dept. Iowa A. C. (Dr. Tait Butler), Odebolt, Iowa; Louis A. Thomas, D.V.S., Chi. Vet. Coll. (Dr. Tait Butler), Atlantic, Iowa; Gulian C. Fagan, D.V.S., Am. Vet. Coll. (Dr. W. H. Hoskins), 232 East One Hundred and Sixteenth Street, New York City; A. W. Swedeburg, V.S., Tor. Vet Coll. (Dr. E. S. Walmer), Third Ave and Pennsylvania Ave., S. E., Washington, D. C.; John A. Meyers, D.V.S., Am. Vet. Coll. (Dr. E. S. Walmer), Harrisonburg, Rockingham Co., Va.; S. L. Hunter, V.S., Tor. Vet Coll. (Dr. D. Lemay). Ft. Leavenworth, Kansas; C. Douglass McMurdo, D.V.S., Am. Vet. Coll. (Dr. D. Lemay), Fort Riley, Kansas; W. H. Richards, V.S., Ont. Vet. Coll. (Dr. D. Lemay), 16 West Fifth Avenue, Emporia, Kansas; C. W. Purcell, V.S., Ontario Vet. Coll. (Dr. W. H. Hoskins), Old Orchard Beach, Me.; M. A. Piche, V.S.. Montreal Vet. Coll. (Waugh and Hoskins), Fort Custer, Mont.; Herbert Neher, D.V.S., Am. Vet. Coll. (Dr. C. B. Michener), 350 West Forty-eighth Street, New York City; Olof Schwartzkopff, V.M.D., Royal Vet. Coll., Berlin, (Dr. W. H. Hoskins), St. Anthony Park, Minn; James L. Kidd, D.V.S, Am. Vet. Coll. (Dr. W. H. Hoskins), 102 East Main Street, Lexington, Ky.; D. B. McCapes, V.S., Ont. Vet. Coll. (Dr W. H. Hoskins), Vermillion, South Dakota; W. L. Williams V.S., Montreal Vet. Coll. (Dr. W. H. Hoskins), Bloomington, Ill.; J. M. Tye, V.S., Ontario Vet. Coll. (Dr. W. H. Hoskins). Muncie, Ind.; A. F. Schreiber, Vet. Dept. U. of Pa. (Dr. W. H. Hoskins), Sixty-first and Elmwood Ave., Philadelphia, Pa.; A. S. Barnes, V.S., Ont. Vet. Coll. (Dr. Tait Builer), Maquoketa, Iowa; T. A. Brown, D.V.S., Chi. Vet. Coll. (Dr. Tait Butler), Chariton, Iowa; J. T. Kennedy, V.S., Ont. Vet. Coll. (Dr. Tait Butler,), West Union, Iowa; H. N. Waller, V.S., Ont. Vet. Coll. (Dr. Tait Butler), Windom, Minn.; Alexander Plummer, D.V.S., Chi. Vet. Coll. (Dr. Tait Butler), Mammoth Hot Springs, Wyoming; S. S. Baker, D.V.S., Chi. Vet. Coll. (Pres. Williams), 609 West Madison Street. Chicago, Ill.; O. J. Lanigan, D.V.S. Chi. Vet. Coll. (Pres. Williams), Wenona, Ill.; J. S. Spangler, D.V.S., Chi. Vet. Coll. (Pres. Williams), Aurora, Ill.; C. E. Sayre, D.V.S., Chi. Vet. Coll. (Pres. Williams), 3725 Cottage Grove Ave., Chicago, Ill.; A. H. Baker, V.S., Mont. Vet. Coll. (Pres. Williams), 2537 State Street, Chicago, Ill.; Joseph Hughes, M.R.C.V.S. (Pres. Williams), 2537 State Street, Chicago, Ill.; R. J. Withers, M.D.V.S. (Pres. Williams), 2537 State Street, Chicago, Ill.; J. M. Wright, Chicago Vet. Coll. (Pres. Williams), Chicago Vet. Co'l., Ill.; G. W. Pope, Chicago Vet. Coll. (Pres. Williams), 2537 State Street, Chicago,

Ill.; R. G. Walker, Chicago Vet. Coll. (Pres. Williams), 2537 State Street, Chicago, Ill.; John Casewell, M.R.C.V.S. (Pres. Williams), 639 W. Madison Street, Chicago, Ill.; P. Quitman, Chicago Vet. Coll. (Pres. Williams), Chicago, Ill.; E. H. Ramsey, Ontario Vet. Coll. (Dr. Paul Paquin), Louisiana, Mo.; L. M. Klutts, Chicago Vet. Coll. (Dr. Paul Paquin), Clinton, Mo.; J. Johnson, Ont. Vet. Coll. (Dr. Paul Paquin), St. Joseph, Mo.; John S. Meyer, Am. Vet. Coll. (Dr. Paul Paquin), St. Joseph, Mo.; Harry B. Piatt, Ont. Vet. Coll. (Dr. Paul Paquin), St. Louis, Mo.; R. J. Sollberger, Berne, Switzerland (Dr. Paul Paquin), St. Louis, Mo.; John W. Conoway, Chicago Vet. Coll. (Dr. Paul Paquin), Columbia, Mo.; F. O'Brien, Ontario Vet. Coll. (Dr. Paul Paquin), Hannibal, Mo.; A. Rouif, Montreal Vet. Coll. (Dr. Paul Paquin), St. Louis, Mo.; C. C. Jackson, Am. Vet. Coll. (Dr. Paul Paquin), Marshall, Mo.; J. Hawkins, Ont. Vet. Coll. (Dr. Paul Paquin), Detroit, Mich.; Wm. Shaw, Ont. Vet. Coll. (Dr. Tait Butler), Dayton, Ohio; C. E. Hollingsworth, Ontario Vet. Coll. (Dr. Williams), La Salle, Ill.; F. H. P. Edwards, Ontario Vet. Coll. (Dr. Tait Butler), Iowa City, Iowa; S. B. Nelson, Iowa Agr. Coll. (Dr. Tait Butler), Ames, Iowa; J. M. Phillips, Chicago Vet. Coll. (Dr. D. Lemay), Wichita, Kansas; E. O. Phillips, Chicago Vet. Coll. (Dr. D. Lemay), Wichita, Kansas; R. Price, Montreal Vet. Coll. (Dr. Lyford), St. Paul, Minn.; Dr. Hinman, Ontario Vet. Coll. (Dr. Lyford), St. Paul, Minn.; T. E. White, Am. Vet Coll. (Dr. Paul Paquin), Sedalia, Mo.

HONORARY MEMBERSHIP.

Prof. J. H. Raymond, M.D, 173 Joralemon Street, Brooklyn, N. Y. First Commissioner of Health to recognize the need of a veterinarian on their staff, proposed by Dr. L. McLean.

Prof. H. M. Biggs, Bellevue Medical College, New York City, foot of E. Twenty-sixth Street, proposed by Dr. H. D. Gill.

LIST OF THOSE DROPPED FOR NON-PAYMENT OF DUES.

Drs. G. S. Agersborg, Vermilion, Dakota; E. C. Beckett, Boston, Mass.; M. Bunker, Newton, Mass.; Joseph Bushman, Washington, D. C.; L. C. Campbell, Philadelphia, Pa.; C. Colburn, West Dedham, Mass.; P. P. Colsson, Mobile, Alabama; J. C. Corlies, Newark, N. J.; J. B. Cosgrove, Worcester, Mass.; L. M. Crane, New York, N. Y.; H. J. Detmers, Columbus, Ohio; W. S. Devoe, New York, N. Y.; William Dimond, Portland, Oregon; G. H. Farnsworth, Rutland, Vt.; S. S. Field, New York, N. Y.; H. T. Foote, New York, N. Y.; John J. Foy, New York, N. Y.; E. Hanshew, Brooklyn, N. Y.; F. J. Hanshew, Brooklyn, N. Y.; William Harris, New York, N. Y.; W. P. Humphrey, Elizabeth, N. J.; James L. Kemp, New York, N. Y.; Robert Laidlaw, Albany, N. Y.; Alex. Marshall, Brookline, Mass.; William R. Mitchell, New York, N. Y.; William Miles, Charleston, Ill.; Samuel W. Mathues, Elam, Pa.; J. E. McNichol, New York, N. Y.; M. J. Otto, New York city, N. Y.; S. L. Richards, Salt Lake City, Utah; J. F. Ryder, Rondont, N. Y.; J. E. Ryder, Jamaica, L. I., N. Y.; J. E. Rice, Hartford, Conn.; Charles Schaufler, Philadelphia, Pa.; William T. Simmons, Boston, Mass.; J. M. Skally, Boston, Mass.; J. M. Walton, New York city, N. Y.; Charles Williams, Philadelphia, Pa.; Charles Winslow, Rockland, Mass.; K. Winslow, Boston, Mass.

DISHONORABLY EXPELLED FOR UNPROFESSIONAL CONDUCT.

Drs. F. S. Billings, Chicago, Ill.; W. B. Prothero, Clearfield, Pa.

DIED BEFORE QUALIFYING

Dr. S. S. Moyer, Hilltown, Pa.

DECEASED.

Drs. A. Lockhart, New York, N. Y.; G. A. Lathrop, Binghamton, N. Y.

RESIGNED.

Dr. A. L. Hummel, Philadelphia, Pa.

